# Impact of diet on cardiometabolic health in children and adolescents

**DOI:** 10.1186/s12937-015-0107-z

**Published:** 2015-11-14

**Authors:** Anna N. Funtikova, Estanislau Navarro, Rowaedh Ahmed Bawaked, Montserrat Fíto, Helmut Schröder

**Affiliations:** 1 Cardiovascular Risk and Nutrition Research Group (CARIN), IMIM (Hospital del Mar Medical Research Institute), Barcelona, Spain; 2 CIBER Epidemiology and Public Health (CIBERESP), Instituto de Salud Carlos III, Barcelona, Spain; 3 Food and Nutrition PhD program, University of Barcelona, Barcelona, Spain; 4 Molecular Oncology Laboratory, Bellvitge Biomedical Research Institute (IDIBELL), Barcelona, Spain; 5 Biomedicine PhD program, University of Pompeu Fabra, Barcelona, Spain; 6 CIBER Physiopathology of Obesity and Nutrition (CIBEROBN), Instituto de Salud Carlos III, Barcelona, Spain

**Keywords:** Diet, Cardiometabolic health, Cardiovascular risk factors, Children, Adolescents

## Abstract

The manifestation of cardiovascular risk factors, such as hypertension, diabetes, and particularly obesity begins in children and adolescents, with deleterious effects for cardiometabolic health at adulthood. Although the impact of diet on cardiovascular risk factors has been studied extensively in adults, showing that their cardiometabolic health is strongly lifestyle-dependent, less is known about this impact in children and adolescents. In particular, little is known about the relationship between their dietary patterns, especially when derived *a posteriori*, and cardiovascular risk. An adverse association of cardiovascular health and increased intake of sodium, saturated fat, meat, fast food and soft drinks has been reported in this population. In contrast, vitamin D, fiber, mono-and poly-unsaturated fatty acids, dairy, fruits and vegetables were positively linked to cardiovascular health.

The aim of this review was to summarize current epidemiological and experimental evidence on the impact of nutrients, foods, and dietary pattern on cardiometabolic health in children and adolescents. A comprehensive review of the literature available in English and related to diet and cardiometabolic health in this population was undertaken *via* the electronic databases PubMed, Cochrane Library, and Medline.

**Electronic supplementary material:**

The online version of this article (doi:10.1186/s12937-015-0107-z) contains supplementary material, which is available to authorized users.

## Introduction

It is well known that atherosclerosis progresses from childhood and adolescence to adulthood [1]. This process is related to the presence of cardiometabolic risk factors such as glucose intolerance, obesity, high blood pressure, high levels of total and low-density lipoprotein (LDL) cholesterol, and low levels of high-density lipoprotein (HDL) cholesterol. Due to the time course of atherosclerosis, it is difficult to establish a direct relationship between risk exposure and cardiovascular disease events; nonetheless, the available evidence indicates that childhood cardiometabolic risk factors are associated with an increased risk of cardiovascular disease in adulthood [1]. Furthermore, cardiometabolic risk variables in childhood are likely to persist into adulthood [2, 3].

Several studies have shown that hypertensive children have increased intimal-medial thickness (IMT) of the carotid artery [4, 5], increased left ventricular mass, and eccentric left ventricular geometry [6]. Data from the longitudinal Cardiovascular Risk in Young Finns Study indicate that childhood blood pressure and serum lipids are strongly related to adult values of these cardiometabolic risk variables [7]. Additionally, intermediate outcomes such as subclinical measures of atherosclerosis are used to determine the association between risk exposure in childhood and cardiovascular disease risk in adulthood. About 60 % of children with elevated blood pressure have hypertension as adults. This persistence of elevated blood pressure has been associated with the highest risk of increased carotid IMT [8]. Findings from the Muscatine, Cardiovascular Risk in Young Finns, and Bogalusa studies revealed an association between childhood cardiovascular risk factors and adult carotid IMT [9–12].

The increasing obesity epidemic has a particularly detrimental effect on cardiometabolic health in children and adolescents. A recent meta-analysis showed that obese children had a higher risk of an adverse cardiometabolic profile, compared to normal weight children [13]. Furthermore, the risk is especially high when overweight or obesity is maintained from childhood to adulthood [14].

High diet quality is strongly related to cardiometabolic health in adults [15, 16]. The Prevention with Mediterranean Diet study (PREDIMED) demonstrated the protective effect of the Mediterranean diet on cardiovascular disease in older patients with high cardiometabolic risk [17]. This review will focus on childhood diet and its role in the development of cardiovascular risk factors. Early prevention, ideally in childhood, could be the best strategy to avoid incidence of cardiometabolic risk factors and premature mortality; conversely, the adoption of a healthy diet at young ages is crucial for disease prevention. We will provide an overview of several aspects of the diet: main nutrients, certain foods known to play a role in cardiovascular health, and the most studied dietary patterns. Results of included studies are summarized in the Additional file 1: Table S1.

### The impact of diet on cardiometabolic health

#### Na and salt intake

Most of the popular snacks that are very attractive to children contain a large amount of salt. In children, the daily recommended Na intake increases with age. For children younger than one year, the daily recommended salt intake is < 1 g/d (range 0.4−1.3 g/d); newborns and infants need more salt per kg of body weight than older children, in whom the adverse effects from excessive salt consumption are similar to adults. In children aged 1 to 5 and 5 to 10 years, the recommended daily intake is 2 g/d and 4 g/d, respectively; however, the actual salt intake reaches 4.9 g/d and 8.1, respectively. For those aged 10 to 20 years the recommendation is 5 g/d, although actual daily intake ranges from 6.7 to 11.0 g/d [18].

He and MacGregor (2006) [19] published a meta-analysis of randomized clinical trials that investigated the effect of reducing salt intake on blood pressure in children and infants. They showed that a reduced salt consumption (median reduction of 42 % in children and 54 % in infants) led to a significant decrease in blood pressure values:–1.17 mmHg (95 % CI–1.8,–0.56 mmHg) systolic and–1.29 mmHg (95 % CI–1.9,–0.65 mmHg) diastolic in children and–2.47 mmHg (95 % CI–4.0,–0.94 mmHg) systolic in infants. More recent studies confirmed those findings [20–22]. Finally, a study carried out in low-income children aged 3–4 years found a higher risk of elevated systolic blood pressure in those who consumed >1200 mg of sodium/day (3.32, 95 % CI 0.98, 11.2) or had >0.5 waist-to-height ratio (8.81, 95 % CI 2.1, 36.3) [23]. However, in other studies, no association was found between excessive consumption of sodium (i.e., exceeding recommended levels) and future high blood pressure [24, 25].

#### Fatty acids, nuts, and olive oil

Fatty acids and fats are an important source of energy, and fat makes food more attractive and tasty, especially for children; however, many studies have shown the positive association between fat consumption and obesity [26, 27]. In clinical and observational studies in children, higher consumption of total, unsaturated and saturated fats, and myristic fatty acids was associated with increased total cholesterol [28, 29]. Thorsdottir and Ramel (2003) found that total and saturated fat consumption was associated with incidence of diabetes [30]. Another randomized trial showed that milk low in saturated fatty acids and enriched in omega-3 polyunsaturated fatty acid (PUFA) and oleic acid reduces indices of endothelial cell activation in children aged 8–14 years [31]. In infants, intake of total fat and monounsaturated fats correlated with apolipoprotein A1 (Apo-A1), the main apolipoprotein of HDL-cholesterol, which is responsible for the efflux of cholesterol from the body (rho = 0.18, *p =* 0.036 and rho = 0.17, *p =* 0.048, respectively) [32]. The intake of polyunsaturated fatty acids was inversely correlated with apolipoprotein B (Apo-B), the main apolipoprotein of LDL-cholesterol and a marker of cardiovascular disease (rho = −0.17, *p =* 0.046).

Few studies have investigated the association of cardiovascular risk factors in children with consumption of food items and food groups rich in lipids. Haro-Mora et al. (2011) showed that children consuming only olive oil, among all vegetable oils used in the study, had lower risk of increased body mass index (BMI) Z-scores (OR 0.19 95 % CI 0.04, 0.52), compared with children consuming a combination of other oils [33]. High nut consumption (>1/4 oz. per day) in children 12–18 years old was associated with lower prevalence of overweight and obesity and lower levels of diastolic blood pressure [34]. A 40 % lower risk of overweight (95 % CI 0.43, 0.85) was observed in the top tertile of nuts consumption among healthy children and adolescents attending Seventh Day Adventist schools, where a high proportion of students are vegetarians or vegans in accordance with religious beliefs [35]. Among food groups with lipid-rich content, vegetable oils were associated with low fasting glucose (*β =* −3.34, 95 % CI–4.1,–0.27) and added fats (cream, butter, lard, creamy dressing, and sauces) were positively associated with higher levels of triglycerides (*β =*2.70, 95 % CI 0.29, 23.3) [36].

#### Dairy

Recently, more attention has been paid to the association between dairy products and cardiovascular risk factors in children. Bigornia et al. (2014) showed that 10-year-olds with higher consumption of full-fat and reduced-fat dairy products had 43 % (95 % CI 0.34, 0.94) and 26 % (95 % CI 0.43, 1.3) lower probability of being overweight or having excessive body fat in 3 years, respectively. [37]. Similar results were found in adolescents [38]. In Mexican children, consumption of flavored milk was associated with decreased risk of obesity (O*R =* 0.88, *p =* 0.004); a similar association was observed for whole milk, but only in univariate analysis, and there was no association for skimmed milk [39]. Flavored milks usually have higher energy per unit than non-flavored milk; however, the sugar and fat content of flavored milks differs according to the brand. Interestingly, consumption of skimmed milk was associated with increased adiposity in 2-to 4-year-olds, compared to consumption of full-fat milk (OR 1.64 and 1.63, *p <* 0.001 for 2-year-olds and 4-year-olds, respectively) [40].

Only a few studies have observed a positive association between consumption of milk and dairy products and adiposity in children [41, 42]; the remainder found inverse or no associations in children and adolescents [43]. Most of the studies about milk consumption were done in European populations; however, Lin Lin et al. (2012) found no association between milk or dairy consumption and both general and abdominal obesity surrogates in a Chinese sample of adolescents aged 11–13 years [44]. The researchers explain this difference in outcome, compared to the European population, as a possible confounding by socioeconomic position in European countries.

Although abdominal obesity is also a cardiovascular risk factor, few studies have investigated the association of dairy products consumption with this type of obesity. In a study by Abreu et al. (2012), high milk consumption was associated with lower abdominal obesity, independently of physical activity level: even the participants with low levels of physical activity and high milk consumption had lower odds of abdominal obesity (OR 0.412, 95 % CI 0.20, 0.85), compared to highly active adolescents with low milk consumption (OR 0.928, 95 % CI, 0.56, 1.53) [45].

Other metabolic syndrome factors, such as insulin resistance, increased blood glucose levels, and diabetes mellitus 2, have also been inversely associated with dairy consumption [46]. In a study with school children from low-income households in Buenos Aires, higher milk consumption was associated with higher levels of the insulin sensitivity marker, homeostatic model assessment (HOMA-IR), independently of other healthy-diet factors (*β =* −0.28, *p =* 0.026) [47]. However, one study of 8-year-old children compared the effect of milk and meat consumption on insulin resistance, and found a positive association between milk (but not meat) consumption and insulin concentration (103 %), insulin resistance (75 %), and C-reactive protein (26 %) [48]. Additionally, in 10- to 16-year-olds from 11 European countries, milk consumption was correlated with incidence of diabetes (*r =* 0.829; *p =* 0.042) [30]. The fat percentage of the milk might also matter; there is a hypothesis that the widespread increase in consumption of low-fat milk and decreased whole milk consumption could be related to an increased inflammation status [49].

Regarding blood pressure, three cohort studies–two in children and one in adolescents–showed an inverse association of dairy consumption with increased blood pressure [43, 50]. Yuan et al. (2013) showed that ≥2 servings per day of dairy products were associated with 1.74 mmHg (*p <* 0.005) and 0.87 mmHg (*p =* 0.010) lower systolic and diastolic blood pressure, respectively, in a fully-adjusted model. In their study, daily servings were defined according to the dairy product: milk, 250 ml; yogurt, 175 g; and cheese, 50 g. They excluded other dairy products, such as ice cream, cream, milkshakes, and combination dishes [50]. However, in school-aged children in Mexico, high intake of high-fat dairy (i.e., produced from whole milk) was associated with higher diastolic blood pressure (*β =* 8.76, 95 % CI 0.75, 2.5) and also with a higher level of HDL-cholesterol (*β =* 10.37, 95 % CI 0.21, 2.0) [36].

### Fruits and vegetables

Many studies have described the beneficial protective association between fruits and vegetables consumption and the development of noncommunicable diseases in children [51]. Although fruits and vegetables are rich in fiber, vitamins, and polyphenols, which makes them a great food for prevention of obesity and other cardiovascular risk factors, many prospective and cross-sectional studies have found no association between fruits and vegetables consumption and childhood obesity [52]. In the last five years, however, some epidemiological and clinical studies [53] have demonstrated a strong protective association between fruits and vegetables consumption and general and abdominal obesity. In school children, fruits and vegetables consumption during school breaks was associated with lower BMI levels [54]. Another study, carried out in school children in the US, found that higher vegetable consumption was associated with 37 % lower odds of being overweight (95 % CI 0.48, 0.94) [35]. More than 3 daily servings of fruits and vegetables were shown to be inversely associated with central adiposity in children [55] and adolescents [56]. In Jamaican adolescents, higher waist circumference (WC) was associated with an absence of fruit consumption (OR 1.75 95 % CI 1.0, 3.0) [57]. The low energy density of fruits and vegetables may explain their protective effect against increased adiposity [58].

More than two servings of fruits and vegetables per day have been associated with reduced blood pressure [59, 60]. The association was even stronger when fruits and dairy products consumption were combined (systolic mean ± SE: 3.03 ± 0.23 (low fruits, vegetables and dairy consumption) vs 1.72 ± 0.45 (high fruits, vegetables and dairy consumption), diastolic mean ± SE: 0.66 ± 0.15 vs 0.25 ± 0.29) [60]. C-reactive protein, a non-specific marker of metabolic disorders and cardiovascular disease, was at the lowest level in children who consumed more vegetables (*p =* 0.0002) [61].

On the other hand, some studies found positive associations between fruits and vegetables consumption and cardiovascular risk factors such as diabetes [30], high glucose [36], obesity [62], central obesity [62], and metabolic risk factors [63]. This could be explained by underreporting of unhealthy foods or overestimation of foods that are perceived as socially desirable and healthy. There is also a hypothesis about the negative impact in Northern European children of increased consumption of tropical fruits, along with a decrease in apples and pears, on inflammation and incidence of type 1 diabetes, probably due to high fructose content [49].

### Vitamins

There is a large gap in the literature on studies of vitamin consumption and the association with cardiovascular risk factors in children and adolescents. The majority of studies are about vitamin D, known to be a strong predictor of type 1diabetes. Other vitamins have received almost no attention from cardiovascular researchers. Therefore, we will necessarily focus on vitamin D and briefly discuss the effect of other vitamins, such as A, E, C, folate, B6 and B12.

### Vitamin D

A recent meta-analysis of 12 cross-sectional studies of international databases showed that vitamin D is inversely associated with level of blood triglycerides (*r =* −0.135, 95 % CI−0.24, −0.03), total cholesterol (*r =* −0.086, 95 % CI–0.02, 0.04), and LDL-cholesterol (*r =* −0.025, 95 % CI–0.22, 0.17) and directly associated with HDL-cholesterol (*r =* 0.156, 95 % CI–0.02, 0.32) in children and adolescents and higher levels of vitamin D are associated with a better lipid profile in children [64]. Higher levels of serum vitamin D was associated with better glucose levels and lipid metabolism and lower general and abdominal adiposity levels, blood pressure, risk of metabolic syndrome, and pubertal development stage in children from different countries [65–74]. In a randomized trial with adolescents, high intake of vitamin D (4000 UI/day) was associated with decreased arterial stiffness, and low intake (200 UI/day) with increased stiffness, but this result was observed in only one of three measurements [75]. Although a recent systematic review showed a lack of consistent evidence for a protective effect of vitamin D against cardiovascular risk factors [76], studies of the association between vitamin D and glucose, diabetes, blood pressure and blood lipids showed inconsistent results.

### Other vitamins

The information about consumption of other vitamins in children and adolescents and its association with cardiovascular risk factors is scarce. Increased consumption of carotenoids, vitamin C and E, was associated with decreased general and abdominal adiposity, impaired metabolism of glucose and lipids, and higher risk of metabolic syndrome [77–79].

There are also some findings regarding B vitamins and their favorable impact on cardiovascular health of children and adolescents. Vitamin B12 and folate were associated with decreased levels of homocysteine (*β =* −0.127 95 % CI −0.24, 0.01; *β =* −0.156 95 % CI–0.29,–0.03) and lower blood pressure in healthy children and adolescents from different countries [80–83].

### Fiber

The effect of fiber intake on cardiovascular health of children and on non-communicable disease in general has not been well studied; even the recommended intake is still under debate [84, 85]. Fiber is a complex nutrient that includes many different compounds, and it has a very strong protective effect against obesity and other cardiovascular risk factors in adults. In light of the modern obesity epidemic among children and adolescents, more attention should be paid to this diet component. Children who eat a high-fiber breakfast have lower insulin resistance and fasting insulin levels in fully adjusted models; however, blood lipids and blood pressure were not affected [86]. In Latino children, higher intake of soluble fiber was associated with lower WC (*β =* 0.069, *p =* 0.036), and participants with no metabolic syndrome traits had significantly higher intake of soluble fiber, compared to children who had 3 metabolic syndrome traits (5.2 vs 4.1 g/day) [87]. In adolescents, total fiber intake was negatively associated with abdominal obesity (*r =* −0.224 for girls, *p <* 0.015;–0.272 for boys, *p <* 0.028) and inflammatory marker plasma C-reactive protein (*r =* −0.230 for girls,–0.308 for boys, *p <* 0.05) [88]. In adolescent girls, fiber intake was positively correlated with percentage of body fat (*r =* 0.22, *p <* 0.01), but this could be due to a high consciousness of healthy food choices among obese girls [89]. Another international study in adolescents showed a positive association of energy-adjusted fiber with percentage of body fat (*β =* 1.7 95 % CI 0.51, 2.9), waist to height ratio (*β =* 0.009 95 % CI 0.00, 0.02) and LDL-cholesterol (*β =* 0.031 95 % CI 0.00, 0.06), but at the same time soluble fiber was inversely associated with serum fasting glucose (*β =* −0.01 95 % CI −0.02, 0.00) [90].

### Cereals and grains

Cereals and grains are the foundation of the plant-based diet, but there are considerably fewer studies about this food group in children and the results are rather consistent. In adolescents and US children, breakfast cereals intake was associated with significantly lower BMI (mean 20.7 vs 21.61, the highest tertile of cereals consumption vs no consumption, *p <* 0.05) and better diet quality; this finding was not dependent on sugar content [91]. In US children, consumption of ≥1.5 servings of grains per day was associated with 40 % lower risk of being obese, in comparison with the lowest quartile of grains consumption, even after adjustment for other dietary predictors such as dairy products, fruits, and vegetables [35, 92]. In US adolescents, higher consumption of whole grains was also associated with lower cardiovascular risk, based on factors such as lower levels of C-peptide (only in girls), fasting insulin, homocysteine (only in boys), higher levels of folates in serum and red blood cells [61, 93], and lower WC (mean of 7.50 vs 6.30 servings/day, WC < 85th percentile vs WC ≥ 85th percentile, *p <* 0.001)) [94]. In a French study, a significant inverse association between obesity and consumption of whole grains and cereals was found in adults, but not in children [95].

### Meat

The impact of meat consumption on cardiovascular health is a subject of debate in both adult and pediatric populations. Study results are not consistent, as the quality and type of meat plays an important role and can change the direction of the association. In Iranian students aged 11 to 18 years, the frequency of red meat consumption was directly associated with dyslipidemia (*β =* 0.04 for total cholesterol, 0.04 for triglycerides,–0.05 for HDL-cholesterol, *p <* 0.05) [96]. In Finnish girls aged 6 to 8 years, the consumption of red meat was associated with higher metabolic risk score, but after adjustment for energy it was no longer significant (*β =* 0.09) [63]. Consumption of poultry, but not red or processed meat, was associated with higher homocysteine levels in adolescents (6.06 [5.8, 6.3] in the 5th quintile vs 5.55 [5.4, 5.8] in the 1st quintile, *p* for trend <0.001) [97]. In another study, US boys from the lowest quartile of central adiposity reported consuming less meat (*p* for trend = 0.025 for children and 0.047 for adolescents), but central adiposity was not related to higher meat consumption [94]. However, in a study of adolescent girls, higher consumption of lean meat was found to be protective against elevated levels of LDL-cholesterol and an unhealthy ratio of LDL to HDL cholesterol [98]. Surprisingly, in Mexican school-age children the consumption of red and processed meat was associated with lower glucose levels (*β =* −7.75, *p =* 0.02) [36], which could be due to selective food energy misreporting (i.e., self-reporting of energy consumption different from actual levels for particular food groups consumed).

Elevated BMI and weight satisfaction [99] are predictors of energy misreporting, which increases with age [100] and has been found to be a common issue among obese participants in a dietary survey [101]. In children, selective energy underreporting (a type of energy misreporting in which reported energy consumption is below the actual level for particular food groups) was associated with higher intake from fruits and vegetables, compared to plausible energy reporters [100].

### Fast food, sugar-sweetened beverages

Several reviews discuss the issue of soft drink consumption and obesity development in children and adolescents, and most of the analyzed studies demonstrate the direct association between this risk factor and the health outcome [102]. Prospective studies revealed a direct association between sugar-sweetened beverages consumption in childhood and future obesity [103]. Studies in children and adolescents from different countries showed a direct association of soft drinks consumption with BMI, waist circumference (WC), overweight, general and abdominal obesity [39, 57, 104–108]. However, consumption of sugar-sweetened beverages was not associated with any obesity measurements in Spanish adolescents [109]. In a recent prospective study in Australian adolescents, increased consumption of sugar-sweetened beverages over time was associated with increased cardiometabolic risk score in girls (OR 3.2 95 % CI 1.6, 6.2), decreased level of HDL-cholesterol in boys and increased level of triglycerides, BMI and WC in both sexes [110]. In Finnish girls aged 6 to 8 years, soft drinks consumption was associated with a higher metabolic risk score (*β =* 0.11, *p <* 0.05) [63]. In a Mexican school-age population, higher consumption of sugar-sweetened beverages was associated with higher diastolic blood pressure (*β =* 6.01, *p =* 0.01) and glucose level (*β =* 7.10, *p =* 0.004) [36]. It is of interest that He et al. (2008) found an association between salt intake and soft drinks consumption in UK children; they showed that 50 % lower salt consumption (approximately 3 g/d less) was associated with a reduced intake of sweetened beverages by approximately 2.3 drinks per week [111].

With fast food, the findings are rather similar to the studies on soft drinks. In UK 13-year-olds, consumption of fast food was associated with increased BMI z-scores (*β =* 0.08, 95 % CI 0.03, 0.14), higher percentage of body fat (*β =* 2.06, 95 % CI 1.3, 2.8), and increased odds of obesity (OR 1.23, 95 % CI 1.0, 1.5) [112]. In Lebanese children, high consumption of fast food also was associated with 3 times increased risk of being overweight (95 % CI 1.2, 8.7) in comparison with low consumption [113]. However, Poti et al. (2014) studied the diet of US children and adolescents after excluding fast food from their analysis, and discovered that the Western diet is probably more responsible for the association with overweight and obesity than the fast food consumption itself (*β =* 5.9, 95 % CI 1.3, 10.5) [114]. Sugar-sweetened beverages, fast food, and various cardiovascular risk factors have been the focus of other studies, most of them with similar results and association trends that show a negative impact on cardiovascular health. These food items are also widely studied within the framework of dietary patterns, particularly the Western dietary pattern, defined by post-hoc analysis.

### Dietary patterns

#### Post-hoc dietary patterns

Many studies in children and adolescents from different countries have analyzed the association of dietary patterns with cardiovascular risk factors. The main dietary patterns derived in those studies are Western or Unhealthy patterns (normally characterized by higher consumption of red meat, meat derivatives, sweets, pastries, fast food, sugar-sweetened beverages, fried foods, and snacks) and Healthy or Traditional patterns (usually distinguished by increased intake of plant-based foods and fish). A majority of these studies showed positive associations between the Western dietary patterns and cardiovascular risk factors, such as obesity and increased triglycerides, and between the Traditional pattern and a healthier cardiovascular profile [115]. However, not all Traditional dietary patterns are associated with better health. Joung et al. (2012) showed that the traditional Korean “Rice & Kimchi” pattern was associated with increased triglycerides and decreased HDL-cholesterol in comparison with other dietary patterns, such as “Noodle & Mushroom” and “Bread & Meat & Fruit & Milk” [115]. Many studies analyzed the association of dietary patterns with adiposity, and confirmed a positive association of a Western dietary pattern and an inverse association of a Healthy dietary pattern with general and abdominal adiposity [114, 116–126]. However, in Scottish children and adolescents, no association was established between healthy or unhealthy patterns and obesity [127].

Other cardiovascular risk factors have also been widely studied. In Mexican [128] and Greek [129] children, analogs of the Western pattern were associated with insulin resistance, with 1.92 and 2.51 higher odds than in the Healthy pattern, respectively. Several studies have paid special attention to metabolic syndrome in children. In Australian and Korean children, the Western dietary pattern in girls was associated with increased risk of metabolic syndrome, and the Healthy pattern was associated with improved glucose and lipid metabolism [118, 130]. In Ecuadorian adolescents, two patterns emerged: “rice-rich non-animal fat pattern” and “wheat-dense animal-fat pattern”, both of them were associated with several cardiovascular risk factors. The first was associated with increased glucose levels in urban adolescents (*p* for trend < 0.01) and the latter with increased levels of total (*p* for trend 0.02) and LDL-cholesterol (*p* for trend 0.04) among rural participants [131].

#### A priori dietary patterns

Significantly fewer studies have used a dietary index, compared to post-hoc dietary patterns. A fairly recent review by Lazarou and Newby (2011) found only 38 studies published through mid-2010 that analyzed the diet-disease relationship using a dietary index in children and adolescents of developed countries [132]. Most of those studies (22, or 57.9 %) were looking at the association between the index scores and anthropometric measurements; only 13 of the studies found a small but statistically significant inverse association between the index values and BMI; almost half of the papers analyzing health outcomes showed an association with such cardiovascular risk factors as high blood pressure, blood lipids, and inflammation markers. It is important to highlight that all studies using a dietary index had a cross-sectional design. In UK children, better scores on a Diet Quality Index and Healthy Diet Indicator were associated with lower body fat (−5.1 %, *p =* 0.023 and–4.9 %, *p =* 0.026, between 1st and 5th quintiles, respectively) and WC (−3.0 %, *p =* 0.005 and–2.5 %, *p =* 0.033, respectively) and other surrogate obesity markers; this was not the case for the Mediterranean Diet Score [133]. However, in an Australian study that validated the Dietary Guideline Index for Children and Adolescents, this index showed a weak but positive association with BMI Z-scores (*β =* 1.13 95 % CI 0.32, 2.0 for 4-to 7-year-olds and *β =* 1.12 95 % CI 0.42, 1.8 for 16- to 18-year-olds) [134].

### Vegetarian diet and mediterranean diet

Few studies have considered a vegetarian diet and the health of children and adolescents. Sabate and Wien (2010) showed that most of the available studies were carried out in the Seventh Day Adventist population, in which a large percentage of people follow plant-based dietary patterns, ranging from vegetarians, who consume dairy foods and eggs, to vegans, who avoid all animal products. Results showed that vegetarian children and adolescents have lower BMI, WC, and LDL-cholesterol and higher HDL-cholesterol [135]. However, not all studies showed a clear inverse association of obesity with cardiovascular risk factors, and some of them did not reach statistical significance in the association with a plant-based diet, but they found a direct inverse association between animal products consumption and the health outcomes studied. It is important to note that in many studies the vegetarian diet was inversely associated with obesity surrogate markers, independently of physical activity. Robinson-O’Brien et al. (2009) also confirmed that vegetarian adolescents, but not children, were less obese (*p <* 0.007) or overweight (*p <* 0.044) than non-vegetarians, but at the same time, extremely unhealthful weight-control behavior (*p <* 0.001) and binge eating (*p <* 0.001) was more popular among vegetarian adolescents than among their non-vegetarian peers [136].

More studies of the diet-disease relationship have analyzed the Mediterranean diet, another traditional plant-based dietary pattern, than vegetarian diet (s). One study in children showed an inverse association between adherence to the Mediterranean diet, measured by the KIDMED index, and arterial stiffness, which is an indicator of higher risk of developing cardiovascular diseases (*β =* −0.114, *p =* 0.026) [137]. In the Mediterranean adolescent population in Balearic Islands, higher adherence to the Mediterranean diet was associated with decreased prevalence of metabolic syndrome and the associated risk factors. However, it was also associated with lower levels of HDL-cholesterol in girls (OR 0.25, 95 % CI 0.05, 0.27, first vs last quartiles) [138]. In an international study in European children, a high food-frequency-based Mediterranean Diet Score (fMDS) was associated with lower risk of overweight/obesity (OR 0.85, 95 % CI 0.77, 0.94, lower fMDS vs higher fMDS) and percentage of fat mass (*β =* −0.22, 95 % CI–0.43,–0.01) independently of physical activity. Furthermore, the fMDS was associated with lower levels of obesity surrogate markers (BMI, WC and waist-to-height ratio) in prospective analysis [139]. Surprisingly, high adherence to the Mediterranean diet was associated with higher salt intake. Every unit increase in the KIDMED index was associated with 10 % increase of sodium intake above the median, >1500 mg/day (95 % CI 1.1, 1.1). The authors clarify that the main source of salt was bread, cheese, and breakfast cereals, which form part of the traditional Mediterranean diet [140]. Other studies found no prospective or cross-sectional association between Mediterranean diet score and cardiovascular risk factors in children [141, 142]. In a non-Mediterranean (UK) population of children, the Mediterranean Diet Score showed no association with obesity or overweight [133]. The few available studies about Mediterranean and vegetarian diets in children and adolescents highlight the favorable and promising role of plant food based dietary patterns, as a complex of healthy food choices, in prevention of cardiovascular risk factors in this population.

## Conclusion

Although most of the studies in children have shown results similar to the analogous studies in adults, many topics studied in adults have not been addressed in children. Vitamins (except vitamin D), polyphenols, and fiber are areas for future development of nutrients research. Food groups such as dairy, fast food, and soft drinks have been studied more extensively than others, but even in these groups, the results are inconsistent. Dietary patterns are a relatively new topic of research, with very few studies in children, both a priori and *a posteriori*. A special gap exists in studies of a priori dietary patterns - very little research has been done on vegetarian diets, the Mediterranean diet, and dietary indexes. Underreporting of foods perceived as unhealthy and overreporting of those perceived as healthy is another problem that results in errors in the analysis of associations between cardiovascular risk factors and foods such as fruits and vegetables. This topic is still not very clearly understood in the adult population, and is even less studied in children. Future studies should focus on the diet’s mechanism of action and on establishing recommendations for healthy dietary habits in children, taking into account age, ethnicity, and health status. Additionally, there is a clear need for interventional studies, not only related to childhood obesity, which is already well developed, but focused on other cardiovascular risk factors as well.

## Additional file

Additional file 1: Table S1. Effect of diet on cardiovascular risk factors in children and adolescents. (DOCX 232 kb)

## Abbreviations

25 (OH) D: 25-hydroxy vitamin D test
Apo-A1: apolipoprotein A1
Apo-B: apolipoprotein B
BMI: body mass index
FMDS: food-frequency-based mediterranean diet score
HDL: high-density lipoprotein
HELENA: healthy lifestyle in Europe by nutrition in adolescence
HOMA-IR: homeostatic model assessment
IMT: intimal-medical thickness
LDL: low-density lipoprotein cholesterol
PREDIMED: prevention with mediterranean diet
PUFA: polyunsaturated fatty acid
WC: waist circumference
