# Supplementary material for: Impact of diet on cardiometabolic health in children and adolescents
Source: Nutr J. 2015 Nov 14;14:118. doi: 10.1186/s12937-015-0107-z (PMC4647337; doi:10.1186/s12937-015-0107-z)
Supplement: Additional file 1: Table S1. — Effect of diet on cardiovascular risk factors in children and adolescents. (DOCX 232 kb) [file 12937_2015_107_MOESM1_ESM.docx]

| **Additional file 1: Table S1. Effect of diet on cardiovascular risk factors in children and adolescents.** | | | | | | | |
| --- | --- | --- | --- | --- | --- | --- | --- |
| **Particular food, nutrient or dietary pattern** | **Study** | **Type of study** | **Duration of the study** | **Number of participants** | **Age** | **Intervention** | **Main outcome** |
| Sodium | Kelishadi et al. Int J Prev Med, 2013[[26](#_ENREF_26)] | cross-sectional |  | 240 | 3-10 years old |  | No association was found between excessive consumption of sodium and future high blood pressure |
| Salt | He et al. J Hum Hypertension, 2008[[21](#_ENREF_21)] | cross-sectional |  | 1658 | 4-18 years old |  | An increase of 1 g/day in salt intake was related to an increase of 0.4 mm Hg in systolic and 0.6 mm Hg in pulse pressure |
| Salt | Yang et al. Pediatrics, 2012[[22](#_ENREF_22)] | cohort | 5 years | 6235 | 8-18 years old |  | 1 g/d of salt intake was associated with 1.0 mm Hg increase in systolic blood pressure among all participants and 1.5 mm Hg increase among overweight/obese participants |
| Sodium | Brion et al. Eur J Clin Nutr, 2008[[25](#_ENREF_25)] | cohort | from infancy to 7 years | 533 with sodium data at 4 months old; 710 with sodium data at 8 months old | 4 and 8 months |  | No association was found between excessive consumption of sodium and future high blood pressure. Majority of infants at 8 months exceeded the recommended levels of sodium intake |
| Sodium | Rosner et al., Hypertension, 2013[[24](#_ENREF_24)] | cohorts | 1988–1994 and 1999-2008 | 3248 in 1988–1994; 8388 in 1999–2008 | 8–17 years old |  | Children, who consumed >3450 mg of sodium/day per 2000 kcal, had 1.36 (95% CI 1.0, 1.8) times higher risk of high blood pressure than those who consumed <2300 mg |
| Salt | Shi et al. Br J Nutr, 2014[[20](#_ENREF_20)] | cohort | 6-22 years | 435 | 4-18 years old |  | 1 g/d increase in salt intake was associated with a 0.2 mm Hg increase in systolic blood pressure |
| Sodium | Vitolo et al., Eur J Pediatric, 2013[[23](#_ENREF_23)] | Randomized Clinical trial | 1 year | 331 | 3-4 years old |  | Children had higher risk of elevated systolic blood pressure if they consume >1200 mg of sodium/day (3.32, 95 %CI 1.0, 11.2) or had >0.5 waist-to-height ratio (8.81, 95 %CI 2.1, 36.3) |
| Salt | He et al Hypertension, 2006[[19](#_ENREF_19)] | Meta-analysis of controlled trials: ten trials of children and adolescents, three trials of infants | Children: 2 weeks to 3 years Infant median duration: 8 weeks to 6 months | 966 children and adolescents, 551 infants | children and adolescents 13 to 16 years, infants | Reduce salt intake compared to control group or group period Median salt reduction of 42% in children and 54 % in infants | In children decrease blood pressure: systolic: -1.17 mm Hg (95% CI -1.8, -0.6); diastolic: -1.29 mm Hg (95% CI -1.9, -0.7). In infants decrease systolic blood pressure: -2.47 mm Hg (95% CI -4.0, -0.9) |
| Saturated fat | Thorsdottir and Ramel, Ann Nutr Metab, 2003[[29](#_ENREF_29)] | cross-sectional |  | 4701 | 10-16 years old |  | Consumption of total and saturated fat was associated with incidence of diabetes type 1 |
| Nuts | O'Neil et al., Nutr Res, 2012[[35](#_ENREF_35)] | cross-sectional |  | 24385 | 3 age groups: 2-11, 12-18 and 19+ years old |  | Individuals that consumed > 7 grams of nuts/day had better nutrient intake and diet quality than did no consumers. Children age 12-18 years old who consumed > 7 grams of nuts had lower prevalence of overweight, obesity and diastolic blood pressure |
| Nuts | Matthews et al. Nutr J, 2011[[36](#_ENREF_36)] | cross-sectional |  | 1764 | 6-19 years old |  | Children and adolescents in the third tertile of nuts consumption had 40% lower risk of overweight (95%CI 0.4, 0.9) |
| Vegetable oil | Perichart-Perera et al., J Am Diet Assoc, 2010[[37](#_ENREF_37)] | cross-sectional |  | 228 | 9-13 year old |  | Vegetable oils were associated with low fasting glucose (β=-3.34, 95% CI -4.1; -0.3) and added fats were positively associated with triglycerides (β =2.70, 95% CI 0.3; 23.3) |
| Saturated fat | Royo-Bordonada et al., Public Health Nutr, 2006[[33](#_ENREF_33)] | cross-sectional |  | 1112 | 6-7 years old |  | High intake of saturated fatty acids (>14.5% of total energy intake) was associated with lower levels of Apo-A1 and HDL-cholesterol and a lower LDL-cholesterol/HDL-cholesterol ratio |
| Olive and other oils | Haro-Mora et al., Eur J Endocrinol, 2011[[34](#_ENREF_34)] | cohort | One year | 92 | 13 years old |  | Children consumed only olive oil had lower risk of increased BMI-Z scores( OR=0.19 95%CI 0.04, 0.52) compared to children consuming other oils |
| Monounsaturated and polyunsaturated fatty acids | Hoppu et al., Nutr Metab Cardiovasc Dis, 2013[[32](#_ENREF_32)] | Randomized clinical trail | From Mother first trimester pregnancy to child 4 years old | 256 mothers participated in the study at the first trimester of pregnancy, and 208 mothers and their infants participated at the one-year study visit and 185 and 127 at 2 and 4 years, respectively. |  | the participants were randomly assigned to three study groups: dietary counselling groups with probiotics (diet/probiotics) or with placebo (diet/placebo) and a control group (control/placebo) | In infants, total fat and monounsaturated fatty intake correlated with Apo-A1 (rho = 0.18, p = 0.036 and rho = 0.17, p = 0.048, respectively). The intake of polyunsaturated fatty acids was inversely correlated with Apo-B (rho= -0.17, p = 0.046) |
| Saturated fat | Niinikoski et al.Hypertension, 2009[[30](#_ENREF_30)] | Randomized clinical trial | from 7 months to 15 years STRIP* data | 540 healthy infants receiving a low-saturated-fat, low-cholesterol diet and 522 healthy infants in control | 7 months | Low saturated fat and low cholesterol diet fat intake 30% to 35% of daily energy intake saturated: monounsaturated plus polyunsaturated fatty acid ratio of 1:2, and cholesterol consumption less than 200 mg/d | The intervention group with diet low in saturated fats and cholesterol had lower blood pressure compared to the control group (1 mmHg differences) |
| Saturated fat | Romeo et al., Nutr Metab Cardiovasc Dis, 2011[[31](#_ENREF_31)] | Randomized clinical trail | 5 months | Participants intervention group n=52, control group n= 53 | 8-14 years old | Intervention group consumed 0.6 L/day enriched dairy product containing EPA and DHA, oleic acid, carbohydrates (sugar and honey), vitamins ,minerals and low in saturated fatty acids Control group consumed 0.6 L /day standard whole milk | Enriched milk low in saturated fat reduced indices of endothelial cell activation. |
| Milk | Abreu et al., Pediatr Obes, 2014[[47](#_ENREF_47)] | cross-sectional |  | 1209 | 15 - 18 years old |  | High milk consumption was associated with lower abdominal obesity, independently of physical activity level (OR 0.41 95%CI 0.2, 0.8) |
| Milk | Hirschler et al., J Pediatr, 2009[[49](#_ENREF_49)] | cross-sectional |  | 365 | 10 +/- 2.3 years old |  | Higher milk consumption was associated with higher levels of the insulin sensitivity marker, HOMA-IR, independently of other healthy-diet factors (β = -0.28, p = 0.026) |
| Flavored and normal milk | Beck et al., Nutr, 2014[[41](#_ENREF_41)] | cross-sectional |  | 319 | 8-10 years old |  | Consumption of flavored milk was associated with decreased risk of obesity (OR=0.88, p=0.004); a similar association was observed for whole milk, but only in univariate analysis, and there was no association for skimmed milk |
| Dairy and milk | Wiley et al., Am J Hum Biol, 2010[[44](#_ENREF_44)] | cross-sectional |  | n = 1493 (2-4 years old), n = 2526 (5-10 years old) | 2-4 and 5-10 years old |  | In 2-4 years old children, total dairy were positively related to BMI percentile (β = 0.4 per 100 kJ; p < 0.001). 2-4 years children in the highest quartile of dairy and milk intake had higher BMIs (β = 7.5-8.0; p < 0.01 and β = 6.3–11.8; p < 0.05, respectively). In children of 5–10 years old, in the highest quartile of milk intake had higher BMIs than those in the lowest quartiles (β = 7.1; p < 0.01). |
| Milk | Thorsdottir and Ramel, Ann Nutr Metab, 2003[[29](#_ENREF_29)] | cross-sectional |  | 4701 | 10-16 years old |  | Milk consumption was associated with incidence of diabetes 1 ( r =0.829; p= 0.042) |
| Dairy | Yuan et al., J Acad Nutr Diet, 2013[[52](#_ENREF_52)] | cross-sectional |  | 610 | 8-10 years old |  | Intake of ≥2 servings of dairy per day was associated with 1.74 mm Hg (p < 0.05) lower systolic blood pressure and with 0.87 mm Hg (p = 0.10) lower diastolic blood pressure |
| Dairy | Perichart-Perera et al., J Am Diet Assoc, 2010[[37](#_ENREF_37)] | cross-sectional |  | 228 | 9-13 years old |  | High intake of high-fat dairy was associated with higher diastolic blood pressure (β = 8.76, p = 0.03) but also with a higher level of HDL-cholesterol (β=10.37, p = 0.01) |
| Calcium and dairy | Dixon et al., Obes Res, 2005[[43](#_ENREF_43)] | cross-sectional and cohort | One year | 342 | 4-10 years old |  | Baseline and over one year calcium intake was inversely associated with sum of skinfolds, trunk skinfolds and BMI. Baseline dairy intake did not have statistically significant association with any measure of obesity. |
| Dairy and milk | Lin et al., PLoS One, 2012[[46](#_ENREF_46)] | cohort | 2 years | 5968 | 11 years old |  | No association between milk or dairy consumption and both general and abdominal obesity surrogates |
| Milk | Scharf et al., Arch Dis Child, 2013[[42](#_ENREF_42)] | cohort | 2 years | 10700 | 2 years old |  | Consumption of skimmed milk was associated with increased adiposity, compared to consumption of full-fat milk (OR 1.64 and 1.63, p<0.001 for 2-year-olds and 4-year-olds, respectively) |
| Dairy | Bigornia et al.J Nutr, 2014[[39](#_ENREF_39)] | cohort | 3 years | 5102 | 10 years old |  | Higher consumption of full-fat and reduced-fat dairy products had 43% (95%CI 0.3, 0.9) and 26% (95%CI 0.4, 1.3) lower probability of being overweight or having excessive body fat in three years respectively |
| Milk | Hasnain et al.Child Obes, 2014[[40](#_ENREF_40)] | cohort | 12 years | 106 | 3-5 years old |  | Children with the lowest milk intakes in early childhood had 7.4% more body fat in later adolescence than those with higher intakes (30.0% body fat in tertile 1 vs. 22.6% in tertile 3; p=0.0095). |
| Milk | Hoppe et al., Eur J Clin Nutr, 2005[[50](#_ENREF_50)] | Randomized clinical trial | 7 days | 48 boys | 8 years old | 24 boys were asked to take about 53 g protein daily, 12 boys - 1.5 l of skimmed milk, and 12 other boys - 250 g low fat meat | High milk consumption caused increase in insulin concentration (103%), insulin resistance (75%), and C-reactive protein (26%). Beta cell function increased significantly in milk and meat groups by 86% and 42%, respectively. Fasting glucose decreased in the meat group (p = 0.008) |
| Fruits and vegetables | Abril et al., Food Nutr Bull, 2013[[56](#_ENREF_56)] | cross-sectional |  | 743 | 6-9 years old |  | Fruits and vegetables intake during the school break was associated with low BMI value |
| Vegetables | Matthews et al., Nutr J, 2011[[36](#_ENREF_36)] | cross-sectional |  | 1764 | 6- 19 years old |  | Higher vegetable consumption was associated with 37% lower odds of being overweight (95%CI 0.5, 0.9) |
| Fruits and vegetables | Downs et al., Appl Physiol Nutr Metab, 2008[[57](#_ENREF_57)] | cross-sectional |  | 178 | 9-12 years old |  | Consumption of more than 3 daily serving of fruits and vegetables were inversely associated with central adiposity 0.43 (95%CI 0.2, 0.98) |
| Fruits and vegetables | Al-Hazzaa et al., BMC Public Health, 2012[[58](#_ENREF_58)] | cross-sectional |  | 2906 | 14 -19 year old |  | Abdominal obesity was inversely associated with high and fruit and vegetable intake |
| Fruits | Francis et al., Public Health Nutr, 2009[[59](#_ENREF_59)] | cross-sectional |  | 1317 | 15-19 years old |  | Higher waist circumference was associated with an absence of fruit consumption (OR 1.75, 95%CI 1.0, 3.0) |
| Fruits and vegetables | Damasceno et al., J Clin Nurs, 2011[[61](#_ENREF_61)] | cross-sectional |  | 794 | 12-17 years old |  | Adolescents consume more than >2 serving of fruits and vegetables per day had lower blood pressure value p<0.001,p<0.021 respectively |
| Fruits and vegetables | Perichart-Perera et al., . J Am Diet Assoc, 2010[[37](#_ENREF_37)] | cross-sectional |  | 228 | 9-13 years old |  | Positive association between fruits and vegetables consumption and glucose level (β = 0.71, p = 0.04) |
| Vegetables | Qureshi et al., Nutr Metab, 2009[[63](#_ENREF_63)] | cross-sectional |  | 4010 | 5-16 years old |  | Children consumed more vegetables ( p < 0.001) had the lowest levels of C-reactive protein, a non-specific marker of metabolic disorders and cardiovascular disease |
| Fruits and vegetables | Abreu et al., Public Health Nutr, 2014[[64](#_ENREF_64)] | cross-sectional |  | 1209 | 15-18 years old |  | Fruits and vegetables consumption was positively associated with cardiovascular risk factor obesity and central obesity |
| Fruits and vegetables | Eloranta et al., Eur J Nutr, 2014[[65](#_ENREF_65)] | cross-sectional |  | 512 | 6-8 years old |  | Positive association between fruits and vegetables consumption and metabolic risk factors |
| Fruits and vegetables | Thorsdottir and Ramel, Ann Nutr Metab, 2003[[29](#_ENREF_29)] | cross-sectional |  | 4701 | 10-16 years old |  | There was positive association between diet high in fat and fruits and vegetables with an increased risk of type 1 diabetes , but vegetables and fruits intake alone was not associated with type 1 diabetes |
| Fruits and vegetables | Moore et al., Epidemiology, 2005[[62](#_ENREF_62)] | cohort | 8 years | 95 | 3-6 years old |  | Two or more servings of fruits and vegetables per day was associated with reduced blood pressure, The association was even stronger when fruits and dairy products consumption were combined (systolic mean±SE: 3.03±0.23 vs1.72±0.45, diastolic mean±SE: 0.66±0.15 vs 0.25±0.29) |
| Vitamin D | Williams et al. Heart, 2011[[79](#_ENREF_79)] | cross-sectional |  | 740 - 5609 | 12-19 years old |  | Inverse association of vitamin D with systolic pressure (coefficient 0.068, 95%CI -0.12, -0.02) and a direct relationship with HDL-cholesterol (coefficient 0.101, 95%CI 0.04, 0.16) |
| Vitamin D | Aypak et al., Eur J Pediatr, 2014[[67](#_ENREF_67)] | cross-sectional |  | 168 | 4-16 years old |  | In obese pubertal group , serum 25 (OH) D was associated with lower level of insulin (p=-0.715,p =0.002) and better HOMA-IR (p =-0.656, p=0.006) |
| Vitamin D | Hirschler et al., Clin Chim Acta, 2014[[68](#_ENREF_68)] | cross-sectional |  | 355 | 9.6 ± 2.3 years old |  | deficient serum 25(OH)D in bivariate correlation was associated with decreased levels of HDL-cholesterol and increased LDL-cholesterol, triglycerides, non HDL-cholesterol, Apo B, Apo B/Apo A, insulin, and HOMA-IR |
| Vitamin D | Ha et al., Med Sci Sports Exerc, 2013[[69](#_ENREF_69)] | cross-sectional |  | 310 | children |  | 25 (OH) D serum levels were inversely associated with abdominal obesity , level of total cholesterol in contrast positively associated with accelerometer-based physical activity |
| Vitamin D | Lee et al., J Pediatr Endocrinol Metab, 2013[[70](#_ENREF_70)] | cross-sectional |  | 205 | 7-9 years old |  | 25(OH)D was negatively associated with BMI (β=-0.10, p<0.01), WC (β=-0.28, p<0.01),and body fat mass (β=-0.12, p=0.02) |
| Vitamin D | Lee et al., Nutr Metab Cardiovasc Dis, 2013[[71](#_ENREF_71)] | cross-sectional |  | 1660 | 9 years old |  | 25(OH)D levels were associated with decreased general and abdominal obesity (p< 0.001), triglyceride levels and HDL cholesterol levels |
| Vitamin D | Choi et al., PLoS One, 2014[[72](#_ENREF_72)] | cross-sectional |  | 260 | 15-16 years old |  | In male adolescents 25(OH)D serum level inversely associate with insulin resistance |
| Vitamin D | Chung et al., Public Health Nutr, 2014[[73](#_ENREF_73)] | cross-sectional |  | 1466 | 10-19 years old |  | Serum 25(OH)D levels were associated with fasting glucose , insulin and HOMA-IR |
| Vitamin D | Kelly et al., Arch Dis Child, 2011[[74](#_ENREF_74)] | cross-sectional |  | 85 | 4-18 years old |  | Serum 25(OH)D levels was associated with higher levels of fasting glucose , insulin and HOMA-IR independently of BMI and pubertal development stage |
| Vitamin D | Parikh et al., Diabetes Care, 2012[[75](#_ENREF_75)] | cross-sectional |  | 701 | 14-18 years old |  | Serum 25(OH)D levels was associated with higher levels of fasting glucose , insulin and HOMA-IR, blood pressure, HDL cholesterol and triglycerides |
| Vitamin D | Moreira et al., Metab Syndr Relat Disord, 2014[[76](#_ENREF_76)] | cross-sectional |  | 496 | 15-18 years old |  | Adolescents with the lowest vitamin D intake had higher odds of cardiometabolic risk factors (3.35, 95%CI 1.3, 8.8) |
| Vitamin D | Oliveira et al., Public Health Nutr, 2014[[77](#_ENREF_77)] | cross-sectional |  | 160 | 15-17 years old |  | Lower levels of BMI and waist circumference in comparison with the highest intake group (mean 26.6 vs 21.7 and 77.9 vs 70.1 for BMI and waist circumference, respectively) |
| Vitamin D | Kelishadi et al., Int J Food Sci Nutr, 2014[[66](#_ENREF_66)] | Meta-analysis of cross-sectional studies |  | 25394 | 1-65 years old |  | Higher levels of vitamin D were associated with a better lipid profile, vitamin D was inversely associated with level of blood triglycerides (r=-0.135, 95%CI -0.24, -0.03), total cholesterol (r=-0.086, 95%CI -0.02, 0.04), and LDL-cholesterol (r=-0.025, 95%CI -0.22, 0.17) and directly associated with HDL-cholesterol (r=0.156, 95%CI -0.02, 0.32) |
| Carotenoids | Gunanti et al., J Nutr, 2014[[80](#_ENREF_80)] | cross-sectional |  | 1154 | 8-15 years old |  | Carotenoids have been associated with a lower level of metabolic syndrome (OR 0.39 95%CI 0.2, 0.8), decreased risk of hypertriglyceridemia (OR 0.35 95%CI 0.3, 0.5), overweight (OR 0.57 95%CI 0.4, 0.9), obesity (OR 0.35 95%CI 0.2, 0.5) and other obesity markers , but serum retinol was associated with increased risk of overweight (OR 2.01 95% CI 1.3, 3.2), obesity (OR 2.90 95% CI 1.7, 5.1), and other obesity markers |
| Carotenoids, vitamin C and E | Beydoun et al., J Nutr, 2012[[81](#_ENREF_81)] | cross-sectional |  | 782–4285 | 12-19 years old |  | Carotenoids have been associated with abdominal obesity (OR 0.43 95%CI 0.3, 0.7) and a lower level of C-reactive protein (OR 0.46 95%CI 0.2, 0.9) and HOMA-IR (OR 0.60 95%CI 0.5, 0.8). Vitamin C have a protective effect against metabolic syndrome status (OR 0.21 95%CI 0.1, 0.7) and hyperuricemia (OR 0.64 95%CI 0.5, 0.9). Vitamin E was associated with decreased risk of abdominal obesity (OR 0.41 95%CI 0.3, 0.7) and HOMA-IR (OR 0.64 95%CI 0.5, 0.9); however, it was directly associated with hypertriglyceridemia (OR 3.73 95%CI 2.0, 7.0) |
| Vitamins A and C | Garcia et al., Nutrients, 2013[[82](#_ENREF_82)] | cross-sectional |  | 197 | 6–10.5 years old |  | Vitamin C had direct association with HDL-cholesterol (correlation coefficients 0.15), and an inverse association with waist to height ratio (-0.16), abdominal fat (-0.20), and body fat (-0.23) α-tocopherol:cholesterol ratio was associated with decreased risk of overweight (OR 0.56 95% CI 0.4, 0.9), obesity (OR 0.41 95%CI 0.3, 0.6), waist to height ratio (correlation coefficients -0.44), abdominal (-0.41) and body fat (-0.44), impaired glucose metabolism indicators (HOMAR-IR, insulin), blood lipids (triglycerides, LDL-cholesterol, total cholesterol) and increased HDL-cholesterol (correlation coefficients 0.215). Vitamin A was direct associated with BMI, BMI-for-age, waist to height ratio and abdominal fat (p < 0.05) |
| Vitamin B12 and folic acid | Tamai et al., Am J Hypertens, 2011[[85](#_ENREF_85)] | cross-sectional |  | 418 | 3-6 years old |  | Higher intake of vitamin B12 was associated with lower blood pressure (6.6 mm Hg difference between systolic quartiles, p trend <0.001; 5.7 mm Hg difference between diastolic quartiles, p trend =0.006), folate was associated with decreased systolic blood pressure (4.1 mm Hg, p trend = 0.004). No association between vitamin B6 and blood pressure |
| Vitamin B and folate | Shen et al., Clin Biochem, 2002[[84](#_ENREF_84)] | cross-sectional |  | 1235 | 12-15 years old |  | Vitamin B12 and folate were associated with decreased levels of homocysteine |
| Vitamin B12 | de Moraes et al., Nutrition, 2014[[86](#_ENREF_86)] | cross-sectional |  | 1089 | 12.5-17.5 years old |  | Vitamin B6 was positively associated with serum blood pressure (β = 3.82; 95%CI, 1.5, 6.2) |
| Vitamin B and folate and homocystein | Brasileiro et al., Nutr Hosp, 2005[[83](#_ENREF_83)] | case-control study |  | 239 | 15-19 years old |  | Vitamin B12 and folate were associated with decreased levels of homocysteine |
| High fiber cereal breakfast | Donin et al., PLoS Med, 2014[[89](#_ENREF_89)] | cross-sectional |  | 4116 | 9-10 years old |  | Children who eat a high-fiber breakfast have lower insulin resistance and fasting insulin levels in fully but blood lipids and blood pressure were not affected |
| Soluble fiber | Ventura et al., J Am Diet Assoc, 2008[[90](#_ENREF_90)] | cross-sectional |  | 109 | 10-17 years old |  | Intake of soluble fiber was associated with lower waist circumference (β = 0.069, P = 0.036), and participants with no metabolic syndrome traits had significantly higher intake of soluble fiber in comparison with children who had 3 metabolic syndrome traits (5.2 vs 4.1 g/day) |
| Dietary fiber | Parikh et al., J Clin Endocrinol Metab, 2012[[91](#_ENREF_91)] | cross-sectional |  | 559 | 14-18 years old |  | Total fiber intake was negatively associated with abdominal obesity (r = -0.224 for girls, p<0.015; -0.272 for boys, p<0.028) and inflammatory marker plasma C-reactive protein (r = -0.230 for girls, -0.308 for boys, p<0.05) |
| Dietary fiber | Vagstrand et al., Eur J Clin Nutr, 2007[[92](#_ENREF_92)] | cross-sectional |  | 469 | 16-17 years old |  | In girls fiber intake was positively correlated with percentage of body fat (r=0.22, p<0.01) |
| Dietary fiber | Lin et al., Eur J Nutr, 2014[[93](#_ENREF_93)] | cross-sectional |  | 1804 | 12.5-17.5 years old |  | There was positive association of energy-adjusted fiber with percentage of body fat (β = 1.7, 95%CI 0.5, 2.9), waist to height ratio (β = 0.009, 95%CI 0.01, 0.02) and LDL-cholesterol (β = 0.031, 95%CI 0.00, 0.06), but at the same time soluble fiber was inversely associated with serum fasting glucose (β =-0.01, 95%CI -0.02, 0.01) |
| Ready to eat cereals | Albertson et al., Nutr Res, 2011[[94](#_ENREF_94)] | cross-sectional |  | 9660 | 6-18 years old |  | Consumption of breakfast cereals was associated with significantly lower BMI (mean 20.7 vs 21.61, the highest tertile of cereals consumption vs no consumption, p<0.05) despite of the sugar content |
| Whole grain | Choumenkovitch et al., Public Health Nutr, 2013[[95](#_ENREF_95)] | cross-sectional |  | 792 | 3rd–6th grade |  | Whole grain consumption (≥1.5 servings of grains per day) was associated with 40% lower risk of being obese in comparison with lowest quartile of grains consumption |
| Grains | Matthews et al., Nutr J, 2011[[36](#_ENREF_36)] | cross-sectional |  | 1764 | 6-19 years old |  | Regular daily consumption of grain was associated with lower risk of being overweight |
| Whole grain | Hur and Reicks, J Acad Nutr Diet, 2012[[96](#_ENREF_96)] | cross-sectional |  | 4928 | 12-19 years old |  | Higher whole grain consumption was associated with better cardiovascular risk factors, lower levels of fasting insulin, C-peptide (for girls), homocysteine (for boys), higher levels of folates in serum and red blood cells |
| Grains | Qureshi et al., Nutr Metab, 2009[[63](#_ENREF_63)] | cross-sectional |  | 4010 | 5-16 years old |  | Lower grains intakes was associated with C-reactive protein (p < 0.001) |
| Grains | Bradlee et al., Public Health Nutr, 2010[[97](#_ENREF_97)] | cross-sectional |  | 5564 | 5-16 years old |  | In adolescents higher consumption of grains was associated with lower waist circumference (mean 7.50 vs 6.30, waist circumference<85th percentile vs waist circumference≥85th percentile, p<0.001) |
| Whole grain | Bellisle et al., Br J Nutr, 2014[[98](#_ENREF_98)] | cross-sectional |  | 855 (3–12 years old), 316 (13-16 years old), | 3-16 years old |  | Whole grains and cereals consumption did not have a significant inverse association with obesity |
| Red meat | Kelishadi et al, Prev Med, 2004[[99](#_ENREF_99)] | cross-sectional |  | 2000 | 11-18 years old |  | Higher red meat consumption was directly associated with dyslipidemia (β=0.04 for total cholesterol, 0.04 for triglycerides, -0.05 for HDL-cholesterol, p<0.05) |
| Red meat | Eloranta et al, Eur J Nutr, 2014[[65](#_ENREF_65)] | cross-sectional |  | 408 | 6-8 years old |  | Higher consumption of red meat was associated with higher metabolic risk score, but after adjustment for energy it was no longer significant (β=0.09). It also was associated with higher levels of systolic (β=0.12) and diastolic (β=0.13) blood pressure (p<0.05) |
| Poultry, red and processed meat | Lutsey et al, Am J Clin Nutr 2006[[100](#_ENREF_100)] | cross-sectional |  | 2695 | 15-20 years old |  | Higher consumption of poultry was associated with higher homocysteine levels (6.06 [5.8, 6.3] in the 5th quintile vs. 5.55 [5.4, 5.8] in the 1st quintile, p trend <0.001) and level of serum B6 (p trend 0.001). Serum folate concentration was inversely and serum vitamin B12 positively associated with intakes of red and processed meat (p = 0.008 and p trend 0.001, respectively) |
| Meat | Bradlee et al, Public Health Nutr, 2010[[97](#_ENREF_97)] | cross-sectional |  | 3761 children, 1803 adolescents | 5-11 and 12-16 years old |  | Boys from the lowest quartile of central adiposity reported consuming less meat (p for trend = 0.025 for children and 0.047 for adolescents), but central adiposity was not related to higher meat consumption |
| Red and processed meat | Perichart-Perera et al., J Am Diet Assoc, 2010[[37](#_ENREF_37)] | cross-sectional |  | 228 | 9-13 years old |  | Higher consumption of red and processed meat was associated with lower glucose levels (β = -7.75, p = 0.02) |
| Lean meat | Bradlee et al, J Hum Nutr Diet, 2014[[101](#_ENREF_101)] | cohort | 10 years | 1461 girls | 9-10 years old |  | No adverse effect of higher intakes of lean meat on lipid levels |
| Sugar-sweetened carbonated beverages | Collison et al, BMC Public Health, 2010[[104](#_ENREF_104)] | cross-sectional |  | 9433 | 10-19 years old |  | BMI and waist circumference were positively correlated with sugar-sweetened carbonated beverage consumption (β = 0.10 for both, p < 0.001) in boys |
| Sugar-added beverages | Linardakis et al., BMC Public Health, 2008[[105](#_ENREF_105)] | cross-sectional |  | 856 | 4-7 years old |  | Consumption of sugar-added beverages was associated with increased risk of obesity according to BMI (OR = 2.35, p = 0.023) and waist circumference (OR = 2.07, p = 0.028), when compared to non-consumers with BMI and waist circumference within the normal ranges |
| Fast food and sweetened beverages | Francis et al., Public Health Nutr, 2009[[59](#_ENREF_59)] | cross-sectional |  | 1317 | 15-19 years old |  | Consumption of fast food >3 times per week and >1 bottle of sweetened beverage per day was associated with increased risk of overweight (OR 1.84, 95%CI 1.1, 3.2) and (OR 1.52, 95%CI 1.1, 2.2) respectively. |
| Caloric soft drinks | Gibson and Neate, Int J Food Sci Nutr 2007[[106](#_ENREF_106)] | cross-sectional |  | 1294 | 7-18 years old |  | High intakes of caloric soft drinks (top tertile 396 kJ/day) were associated with increased odds of overweight, compared with low intakes (<163 kJ/day) (OR 1.39, 95%CI 1.0 2.0). The highest quintile of soft drink consumption (>550 kJ/day) was associated with significantly increased odds of overweight (OR 1.67, 95%CI 1.0, 2.7) |
| Soft drinks | Li et al, Asia Pac J Clin Nutr, 2010[[107](#_ENREF_107)] | cross-sectional |  | 1804 | 11-17 years old |  | Consumption of one and two bottles of soft drink a day was associated with increased risk of overweight and obesity (OR 1.5, 95%CI 1.2, 2.4 and OR 1.7, 95%CI 1.1, 2.7 respectively) |
| Soda | Beck et al, Public Health Nutr, 2013[[41](#_ENREF_41)] | cross-sectional |  | 219 | 8-10 years old |  | For each additional serving of soda (240 ml) consumed weekly, the odds of obesity increased by 29 % (95%CI 1.1, 1.5) |
| Sugar beverages | Papandreou et al, Hippokratia, 2013[[108](#_ENREF_108)] | cross-sectional |  | 607 | 7-15 years old |  | Consumption of sugar beverage was significantly associated with overweight and obesity (OR 2.57 95%CI 1.1, 3.4) |
| Sweetened soft-drinks | Gomez-Martinez et al, Nutr Hosp, 2009[[109](#_ENREF_109)] | cross-sectional |  | 1523 | 13-18 years old |  | No difference was observed between the groups of frequency of SSD consumption in any of the anthropometric measurement, BMI or calculated body fat |
| Sugar-sweetened beverages | Eloranta et al, Eur J Nutr, 2014[[65](#_ENREF_65)] | cross-sectional |  | 408 | 6-8 years old |  | Consumption of sugar-sweetened beverages was associated with a higher metabolic risk score (β=0.11, p<0.05) |
| Soft drinks/sweetened beverages | Perichart-Perera et al., J Am Diet Assoc, 2010[[37](#_ENREF_37)] | cross-sectional |  | 228 | 9-13 years old |  | Higher consumption of sugar-sweetened beverages was associated with higher diastolic blood pressure (β = 6.01, p = 0.01) and glucose level (β = 7.10, p = 0.004) |
| Fast food | Nasreddine et al., Nutrients, 2014[[113](#_ENREF_113)] | cross-sectional |  | 868 | 6-19 years old |  | Fast food high consumption was associated with 3 times increased risk of being overweight (95% CI 1.2, 8.7) compared with low consumption |
| Sugar-sweetened beverages | Ambrosini et al, Am J Clin Nutr, 2013[[110](#_ENREF_110)] | cohort | 3 years | 1433 | 14 years old |  | Girls who moved into the highest tertile of sugar-sweetened beverage had 3.8% (95%CI 1.5, 9.3) and 2.7% (95%CI 1.3, 5.6) higher risk of overweight/obesity and being in classified at the metabolic risk, respectively. In comparison with maintaining the lowest tertile of sugar-sweetened beverage, moving into the highest tertile was associated with increase in BMI of 3.8% (95%CI 1.8%, 5.7%) in girls, in systolic blood pressure if 1.7% (95%CI 0.3 ,3.1) in girls, in waist circumference of 2.3% (95%CI 0.7%, 4.0%) in boys and of 4.2% (95%CI 2.5%, 5.9%) in girls, in reduction of HDL-cholesterol of 5.1% (95% CI 1.4%, 8.9%) in girls and 3.8% (95% CI 0.5%, 7.1%) in boys. Moving into the highest tertile of sugar-sweetened beverage intake was associated with increases in triglycerides of 10.8% (95% CI 4.2%, 17.3%; p trend = 0.001) in girls and 10.4% (95% CI 3.4%, 17.5%; p trend = 0.003) in boys. |
| Fast food | Fraser et al., Am J Prev Med, 2012[[112](#_ENREF_112)] | cohort | 4 years | 4827 | 13 years old |  | Fast food consumption was associated with increased BMI z-scores (β = 0.08, 95% CI 0.03, 0.14), higher percentage of body fat (β = 2.06, 95% CI 1.3, 2.8), and greater odds of obesity (OR 1.23, 95% CI 1.0, 1.5) |
| "Noodle & Mushroom", "Bread & Meat & Fruit & Milk", "Rice & Kimchi" dietary patterns | Joung H et al.,Korean J Pediatr[[115](#_ENREF_115)] | 4 Cross-sectional studies, surveys KHANES |  | 3168 | 13-18 years old |  | The risk of elevated serum triglyceride was low in adolescents in the "Noodle & Mushroom" (OR, 0.5; 95% CI 0.3, 0.8) dietary pattern groups, and the risk of low HDL-cholesterol levels was low in adolescents in the "Bread & Meat & Fruit & Milk" pattern group (OR, 0.68 95% CI 0.5, 0.9) compared to the "Rice & Kimchi" pattern group |
| "Western" and "Healthy" dietary patterns | Ambrosini et al., Nutr Metab Cardiovasc Dis, 2010[[118](#_ENREF_118)] | cross-sectional |  | 1139 | 14 years old |  | The "Western" dietary pattern in girls was associated with increased total cholesterol (p trend = 0.03), augmented the risk of being in the “high risk metabolic cluster” (OR 2.50 95%CI 1.1, 6.0, the 4^th^ quartile vs the 1^st^ quartile) and increases mean WC (p trend = 0.03) and BMI (p trend = 0.02). The "Healthy" pattern was associated with decreased glucose level in girls and boys (p trend = 0.01 and 0.04, respectively) and increased HDL-cholesterol in boys (p trend = 0.02). |
| "Treat Foods", "Fruits and Vegetables", and "Basic Foods" | Howe et al., Nutrition J, 2013[[119](#_ENREF_119)] | cross-sectional |  | 681 | 14-18 years old |  | Every SD increase in the ‘Basic Foods’ score was associated with decrease of 3.58% (95% CI −6.14, -0.94) in the geometric mean for fat mass index. With increasing ‘Basic Food’ scores there was a significant decrease in waist circumference, waist-to-height ratio, fat mass index and fat-free mass index for boys |
| Rural, Sweet cereal and corn dishes, Diverse, Western, Whole and sweet dietary patterns | Rodriguez-Ramirez et al, Arch Latinoam Nutr, 2011[[122](#_ENREF_122)] | cross-sectional |  | 8252 | 5-11 years old |  | Sweet cereal and corn dishes and Western dietary patterns had a prevalence ratio of being overweight and obesity of 1.29 (95%CI 1.09, 1.94) and 1.35 (95%CI 1.17, 2.19), respectively, in comparison with the reference, Rural dietary pattern. |
| Traditional food, Healthy food, Fast food dietary patterns | Shang et al., Appl Physiol Nutr Metab, 2014[[123](#_ENREF_123)] | cross-sectional |  | 630 |  |  | ↑ Fast food dietary pattern was associated with overweight (BMI ≥ 85th percentile) and ↑ BMI, waist circumference and body fat mass percentage (p < 0.05). |
| "Healthy", "unhealthy" dietary patterns | Manios et al, Eur J Clin Nutr, 2010[[124](#_ENREF_124)] | cross-sectional |  | 2317 | 1-5 years old |  | One unit increase in "unhealthy" dietary pattern score was associated with almost 11% higher probability of being obese (p = 0.034). Fourth quartile of the "unhealthy" dietary pattern was associated with 30% increased risk of being obese in comparison with the first quartile. |
| "Healthy", "Western", "Sweet-Dairy" dietary patterns | Bahreynian et al, Int J Prev Med, 2013[[125](#_ENREF_125)] | cross-sectional |  | 637 | 7-11 years old |  | In girls: the second quartile of the "Healthy" pattern was more likely to have higher BMI (OR 2.23 95%CI 1.0, 5.0) compared to the second quartile; the second quartile of "Western" dietary pattern had significantly lower BMI than the forth quartile (OR 0.46, 95%CI 0.2, 1.0); lower adherence to "Sweet-Dairy" pattern was associated with lower BMI (OR 0.42, 95%CI 0.2, 0.9). In boys significant association was observed between first and fourth quartile of "Healthy" pattern (OR 0.36, 95%CI 0.2, 0.8). |
| Healthy, transitive, Western dietary patterns | Shang et al, PLoS One, 2012[[126](#_ENREF_126)] | cross-sectional |  | 5267 | 6-13 years old |  | Children with the Western dietary pattern had a significantly higher risk of obesity (OR 1.80 95%CI 1.2, 2.8) compared with children from the healthy dietary pattern. Children with the transitive dietary pattern and children with the Western dietary pattern had significantly higher odds of abdominal obesity, 1.71 (95%CI 1.1, 2.6) and 1.31 (95%CI 1.1, 1.6), respectively. |
| "Fruit and vegetables", "Vegetables", "Fruit", "Puddings", "Snacks", "Fish and sauce", "Starchy food and drinks" dietary patterns | Craig et al, Brit J Nutr, 2010[[127](#_ENREF_127)] | cross-sectional |  | 1233 | 5-11 and 12-17 years old |  | There were no clear association between BMI and dietary patterns. In boys aged 5–11 years, obese children had the lowest factor score in "snacks" dietary pattern (p trend = 0.047). In the "fish and sauce" dietary pattern 5-11 years old obese boys had the highest factor score (p trend 0.023). |
| "Western", "prudent", "high protein/fat" dietary patterns | Romero-Polvo et al, Ann Nutr Metab, 2012[[128](#_ENREF_128)] | cross-sectional |  | 916 | 7-18 years old |  | Participants in the highest quintile of the Western pattern had 92% greater odds of insulin resistance (95%CI 1.1, 3.4) compared with those in the lowest quintile. |
| Fried potato, red meat and sugared beverages; processed meats and cheese; margarine, sweets and savory snacks; legumes and fruits; higher egg and lower fish dietary patterns | Karatzi et al, Public Health Nutr, 2014[[129](#_ENREF_129)] | cross-sectional |  | 1913 | 9-13 years old |  | Margarine, sweets and savory snacks dietary pattern was positively associated with HOMA-IR (β = 0.08, p = 0.001). Children from the third tertile of margarine, sweets and savory snacks dietary pattern were 2.51 (95%CI 1.3, 4.9) times more likely to have insulin resistance compared with children in the first tertile. |
| "Balanced", "western" dietary patterns | Park et al, Nutr Res Pract, 2013[[130](#_ENREF_130)] | cross-sectional |  | 1008 | 8-9 years old |  | Only in girls several metabolic syndrome risk factors were significantly associated with dietary pattern scores: the "western" dietary pattern was associated with increased risk of metabolic syndrome (p trend 0.026), waist circumference (p trend = 0.088) and triglycerides (p trend = 0.074); a "balanced" dietary pattern was associated with decreased triglycerides (p trend 0.032). |
| "Rice-rich non-animal fat", "wheat-dense animal-fat" dietary patterns | Ochoa-Aviles et al, BMC Publ Health, 2014[[131](#_ENREF_131)] | cross-sectional |  | 606 | mean age 13.6 ± 1.2 (±SD) years old |  | The "rice-rich non-animal fat" dietary pattern urban adolescents was associated with a moderate increase in glucose blood levels (p < 0.01), the "wheat-dense animal-fat" dietary pattern in rural participants was associated with an increased total (p = 0.02) and LDL-cholesterol (p = 0.04). |
| At baseline: "Vegetable", "fruit", "starchy food", "sweet & salty snack food" dietary patterns. At follow-up younger adolescents: "vegetable", "fruit", "starchy food", "sweet & salty snack food", "fast food". At follow-up older adolescents: "vegetable & fruit", "starchy food", "sweet & salty snack food", "fast food" | Cutler et al., Brit J Nutr, 2012[[120](#_ENREF_120)] | cross-sectional and cohort | 5 years | 4746 at baseline, 2516 at follow-up | middle age at middle school 12.8 - 12.9 years old; middle age at high school 15.8 - 15.9 years old |  | Cross-sectional analysis at baseline, risk of being overweight/obese: ↑ "vegetable" pattern was significantly associated with lower odds in older girls; ↑ "sweet & salty snack food" pattern was associated with lower odds in the younger and older boys; ↑ "fruit" pattern was associated with higher odds in younger boys. Cross-sectional analysis at follow-up, risk of being overweight/obese: ↑ "vegetable & fruit" pattern was associated with lower odds in older girls. ↑ "vegetable" pattern was associated with lower odds in the younger girls. Prospective analysis of being overweight/obese: ↑ "vegetable" pattern was associated with decreased odds in older girls, ↑ "sweet & salty snack food" pattern was associated with decreased odds in older boys and ↑ "fruit" pattern was associated with increased odds in the younger boys; after adjustment for the weight at baseline, the statistical significance was lost. |
| Snacking, Sweet and fat, Vegetables and whole meal, Protein and water dietary patterns | Pala et al., Eur J Clin Nutr, 2013[[116](#_ENREF_116)] | cohort | 2 years | 14989 at baseline, 9427 at follow-up | 2-10 years old |  | Only Vegetables and whole meal was associated with lower risk of overweight/obesity (OR 0.69 95%CI 0.5, 0.9 third tertile in comparison with first). High intake of Vegetables and whole meal was associated with smaller increment in BMI (0.73 vs 0.80 kg/m2 for the second vs first tertile of intake; p=0.04) |
| Snacking dietary pattern | Shroff et al., Public Health Nutr, 2014[[117](#_ENREF_117)] | cohort | 2.5 years | 961 | 5-12 years old |  | Highest quartile of the snacking pattern had a 0.09 kg/m2 per year higher BMI gain than in the lowest quartile (p trend = 0.05). Highest quartile of subscapular: triceps skinfold thickness ratio had a 0.012/year greater increase compared with the lowest quartile (p trend = 0.05). Only soda intake had positive linear association with adiposity indicators. |
| Health Aware, Traditional and Packed Lunch dietary patterns | Smith et al., Food Nutr Res, 2014[[121](#_ENREF_121)] | cohort | 2 years | 3911 | Diet at 10 years old. Body composition at 9 and 11 years old |  | In girls, ↑ of 1 SD in Health Aware score gave an estimated 1.2% (95% CI: 0.0%, 2.4%) ↓ in fat mass gain, ↑ of 1 SD in Packed Lunch score gave an estimated 1.1% (95% CI: 0.0%, 2.2%) ↓. The Health Aware pattern was associated with 0.3% (95% CI: 0.0%, 0.6%) ↓ in lean mass gain in girls. In boys, an ↑ of 1 SD in Packed Lunch score gave 0.3% (95% CI: 0.1%, 0.5%) ↑ in lean mass gain. |
| The Diet Quality Index, the Healthy Diet Indicator | Jennings et al, J Nutr, 2011[[133](#_ENREF_133)] | cross-sectional |  | 1700 | 9-10 years old |  | In the highest quintile of the Diet Quality Index body weight (-5.9%; p =0.002), BMI (-4.2%; p =0.004), BMI Z-score (-44.6%; p =0.006), waist circumference (-3.0%; P =0.005), waist to height ratio (-2.4%; p =0.014), and percentage of body fat (-5.1%; p =0.023) were lower compared with children in the lowest quintile. In the highest quintile of the Healthy Diet Indicator waist circumference (-2.5%; p =0.033), waist to height ratio (-3.2%; p =0.001), and percentage of body fat (-4.9%; p =0.026) were lower compared with the lowest quintile. Each 1-SD increase in the Diet Quality Index score was associated with body weight (p = 0.008), BMI (p = 0.020), BMI Z-score (p = 0.030), waist circumference (p = 0.017), waist to height ratio (p = 0.036), and percentage of body fat (p = 0.027). In the Healthy Diet Indicator score each 1-SD increase was associated with BMI (p = 0.014), BMI Z-score (p = 0.029), waist circumference (p = 0.012), waist to height ratio (p = 0.001), and percentage of body fat (p = 0.007) |
| The Dietary Guideline Index for Children and Adolescents | Golley et al, J. Nutr., 2011[[134](#_ENREF_134)] | cross-sectional |  | 3416 | 4-16 years old |  | The Dietary Guideline Index for Children and Adolescents was positively associated with BMI Z-scores (β = 1.13 95%CI 0.3, 2.0 for 4- to 7-year-olds and β = 1.12 95%CI 0.4, 1.8 for 16- to 18-year-olds). In the 4- to 7-year old group β for BMI Z-score was 0.005 (95%CI 0.00, 0.01). |
| Vegetarian diet | Robinson-O'Brien, et al., J Am Diet Assoc, 2008[[136](#_ENREF_136)] | cross-sectional |  | 2516 | 15-23 years old |  | In the older cohort, vegetarians had lower BMI (p = 0.007) and were less likely to be overweight (p = 0.012) or obese (p = 0.044) in comparison with never vegetarians. |
| KIDMED index of Mediterranean diet | Lydakis et al, Eur J Pediatr, 2012[[137](#_ENREF_137)] | cross-sectional |  | 277 | 12 years old |  | The KIDMED index was negatively correlated with augmentation index (AI) in the brachial artery (β = -0.114, p = 0.026), used as an index of arterial stiffness. |
| Mediterranean diet score | Mar Bibilone et al, Nutr Metab Cardiovasc Dis, 2011[[138](#_ENREF_138)] | cross-sectional |  | 362 | 12-17 years old |  | The forth quartile of adherence to the Mediterranean diet was associated with a lower level of triglyceride (OR 0.25 95%CI 0.05,1.3) and low HDL-cholesterol (OR 0.42 95%CI 0.14, 1.3) in comparison with the first quartile. |
| KIDMED index of Mediterranean diet | Farajian et al, Atherosclerosis, 2011[[142](#_ENREF_142)] | cross-sectional |  | 4786 | 10-12 years old |  | Adiposity indicators did not differ between the KIDMED score groups |
| Mediterranean Diet Score | Jennings et al, J Nutr, 2011[[133](#_ENREF_133)] | cross-sectional |  | 1700 | 9-10 years old |  | The Mediterranean Diet Score was not associated with weight status. |
| Food frequency-based Mediterranean Diet Score | Tognon et al, Nutr Metab Cardiovasc Dis, 2014[[139](#_ENREF_139)] | cross-sectional and cohort | 2 years | baseline: 16220, follow-up: 9114 | 2-9 years old |  | In cross-sectional analysis high levels of the Mediterranean diet score was associated with decreased risk of overweight/obesity (OR 0.85, 95%CI 0.8, 0.9) and percent fat mass (β = -0.22%, 95%CI 0.4, 0.01). In prospective analysis, the Mediterranean diet score was associated with lower BMI (OR 0.87, 95%CI 0.8, 0.98 the highest adherence at baseline vs. the highest quintile of prospective change), waist circumference (OR 0.87, 95%CI 0.8, 0.98) and waist to height ratio (OR 0.88, 95%CI 0.8, 0.99). |
| Mediterranean Diet Score | McCourt et al, Brit J Nutr, 2014[[141](#_ENREF_141)] | cohort | 9 years | 487 | 12-15 years old |  | Serum homocysteine concentration was highest in the group with the highest adherence to the Mediterranean diet at baseline and the lowest at follow-up, and the lowest homocysteine was in the group with the lowest adherence to the Mediterranean diet at baseline and the highest at follow-up. No other significant results were obtained. |
